# Cryoablation of a Symptomatic Chest Wall Desmoid Tumor Underneath a Silicone Breast Implant

**DOI:** 10.1155/2019/2650790

**Published:** 2019-12-18

**Authors:** Siddhant S. Kulkarni, Amy R. Deipolyi, Yolanda C. D. Bryce, Joseph P. Erinjeri

**Affiliations:** Interventional Radiology Service, Department of Radiology, Memorial Sloan Kettering Cancer Center, New York, NY, USA

## Abstract

Desmoid tumors are locally aggressive tumors that have a high rate of reoccurrence, even after resection. Percutaneous cryoablation is an effective alternative treatment with less associated risk. A patient in the fifth decade of life with a history of ductal carcinoma-in-situ, status post bilateral mastectomy and silicone implant placement, presented with a palpable mass in the left breast, core biopsy proven to be a desmoid tumor underneath the implant. The patient underwent two cryoablation procedures in a six-month period. During both procedures part of the implant was included in the ablation zone without any negative effects on the implant. Cryoablation is a feasible treatment option for desmoid tumors adjacent to silicone breast implants.

## 1. Introduction

Desmoid tumors, also known as aggressive fibromatosis, are locally aggressive tumors with no known potential for metastasis. These tumors however have a high rate of local recurrence even after total resection [1]. Most desmoid tumors occur sporadically, however there are some associations with familial adenomatous polyposis (FAP) syndrome, high estrogen states such as pregnancy, and previous local trauma [2]. Most desmoid tumors present as painless or minimally painful slowly growing masses. These tumors can occur at virtually any body site. Because there are no radiographic characteristics that distinguish desmoid from other soft tissue tumors, diagnosis is made by core biopsy and histological analysis. This case report entails a case where a patient with silicone breast implants underwent cryoablation twice with no adverse events noted.

## 2. Case Report

**Figure 1:**
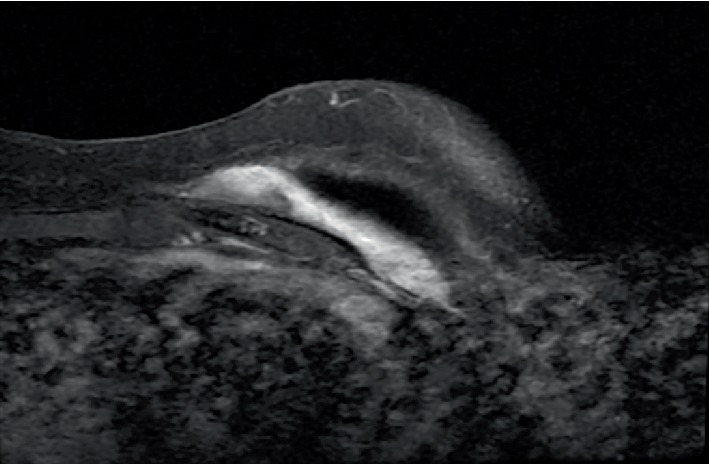
T1-weighted post-contrast MRI of the left breast demonstrates a well demarcated soft tissue mass posterior to the silicone breast implant.

A patient in the fifth decade of life with a history of ductal carcinoma-in-situ (DCIS), status post bilateral mastectomy and silicone implant placement, was referred to interventional radiology for management of a desmoid tumor. Three years after mastectomy the patient felt a mass underneath a left breast implant (Figure 1). Subsequent core biopsy diagnosed the lesion as a desmoid tumor. The patient was initially treated with sorafenib, a tyrosine kinase receptor inhibitor, for one year, with excellent response. Due to toxicity from sorafenib, the patient stopped taking sorafenib for 4 months. The tumor began to grow again and the patient reported constant dull 3/10 chest pain with intermittent 10/10 sharp pain twice daily.

The patient was seen by surgical oncology, but due to the location of the tumor on the anterior chest wall, resection would require partial rib and sternal resection. The patient declined surgery, concerned about poor quality of life after an extensive resection. A plastic surgery consult was obtained, and removal of the silicone breast implant prior to cryoablation was suggested, with replacement after the ablation. Plastic surgery would then replace the implant at a later date. After a multidisciplinary discussion, cryoablation with the implant in place with a short interval follow-up to assess for damage to the implant was preferred, to avoid subjecting the patient to multiple procedures.

**Figure 2:**
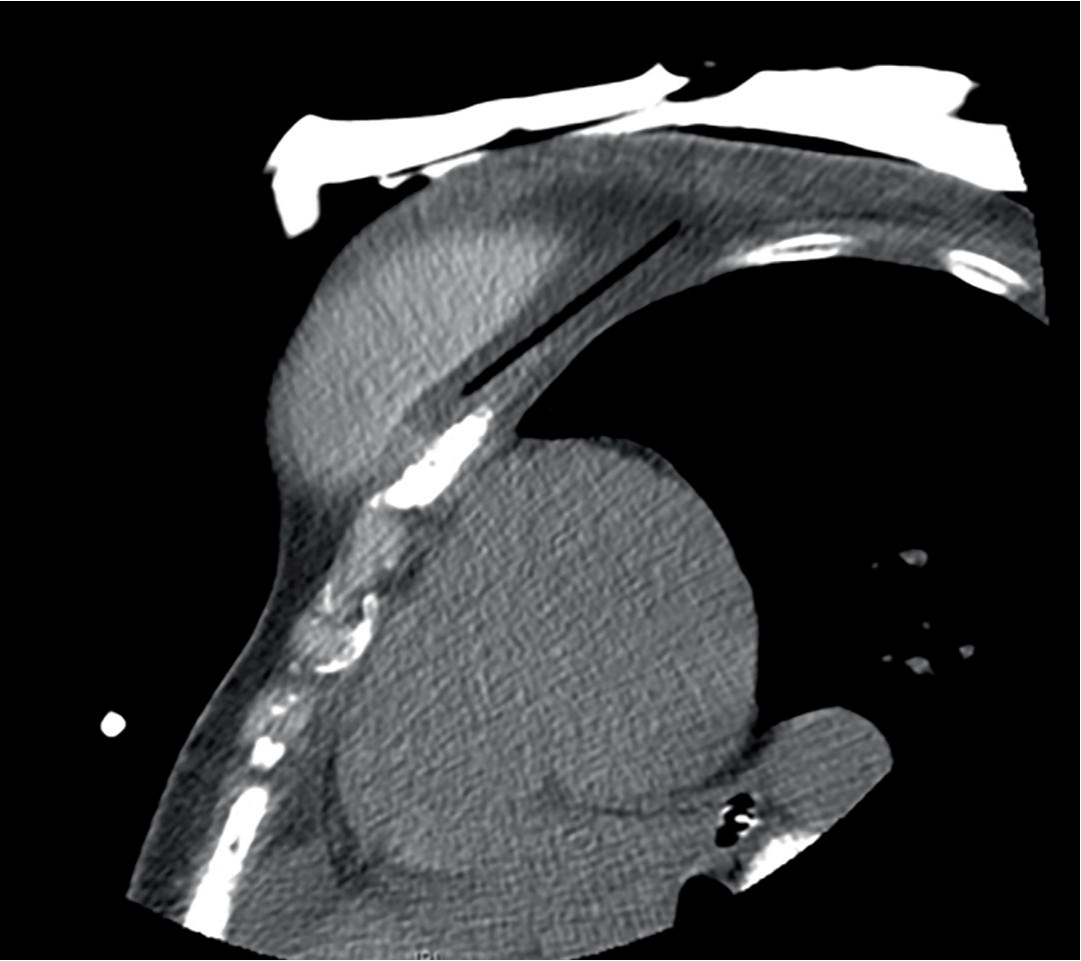
CT image during the second cryoablation demonstrates the cryoprobe's trajectory posterior to the implant. The ice ball extends into the implant.

**Figure 3:**
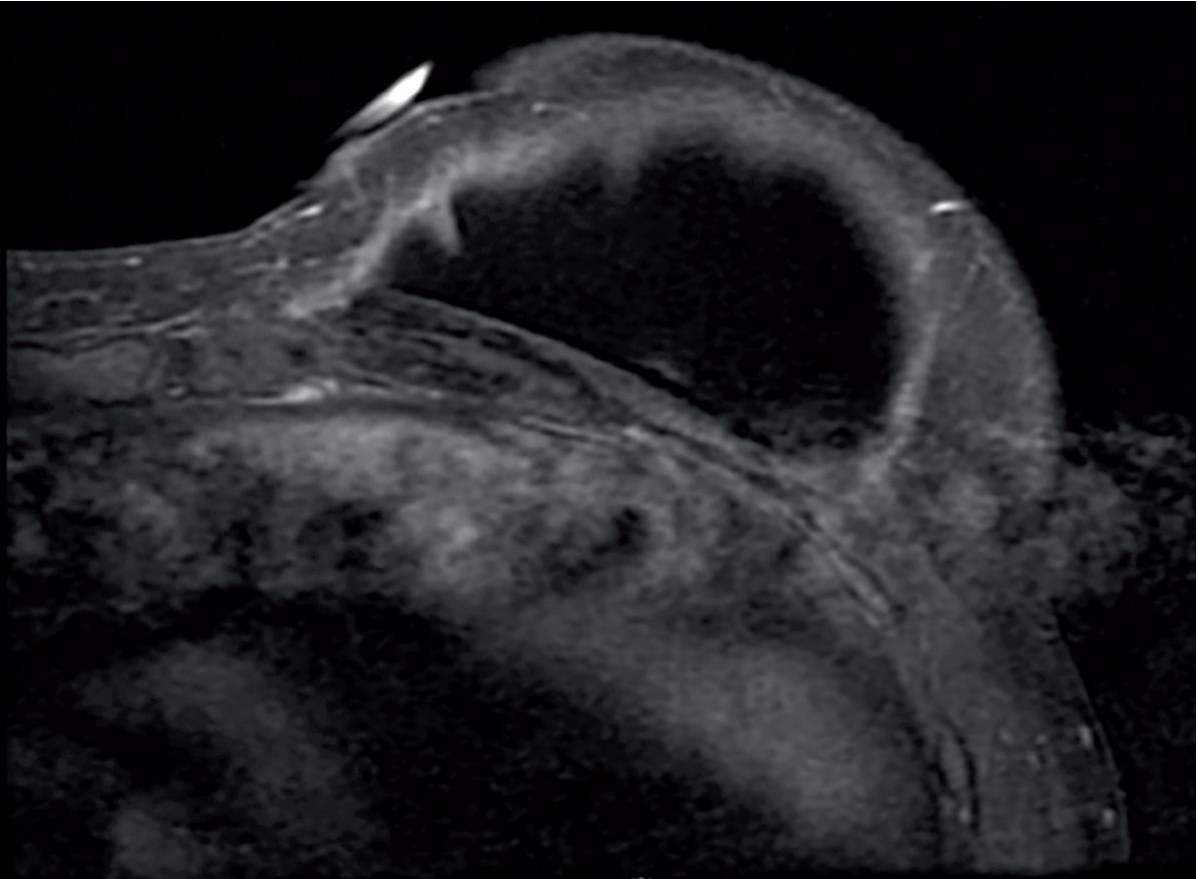
Follow up T1-weighted post-contrast MRI of the breast performed 9 months after the second cryoablation demonstrates reduction of size of desmoid tumor and intact breast implant.

During cryoablation, the patient was positioned supine, left posterior oblique, to minimize the overlap between the expected ablation zone and the implant. Hydrodissection was attempted, but it was not possible to isolate the tumor from the implant. During cryoablation the ice ball encroached approximately one centimeter into the breast implant. After the procedure, the patient's pain improved to 1/10 with no signs of breast implant rupture. Three months later imaging demonstrated a small area of enhancement along the lateral aspect of the desmoid tumor, likely representing small amount of residual tumor, though the patient was asymptomatic. Six months later, the dull pain recurred. Repeat imaging showed increased size of the lateral aspect of the tumor. A second cryoablation was performed with the silicone implant in place (Figure 2). Though the ice ball extended several centimeters into the implant, the integrity of the implant remained intact. Follow up imaging at 9 months demonstrated no residual tumor, and the patient reported complete resolution of pain (Figure 3).

## 3. Discussion

Treatment options for desmoid tumors are dictated by anatomic site of the tumor and include observation, medical treatment, surgical resection with negative margins, radiation therapy, and thermal ablation [3–5]. Cryoablation and radiofrequency ablation are safe and effective treatments for desmoid tumors with few adverse events and recurrence rates similar to traditional therapies [6, 7]. Ablation is also less invasive than surgical resection, with reduced recovery time.

Silicone breast implants have an outer shell filled with silicone gel [8]. Possible injuries to silicone breast implants include rupture, implant wrinkling, implant displacement, extrusion of implant through skin, and capsular contraction around the implant [9]. However, there are no published reports of the results of cryoablating a silicone breast implant and the outcomes of the trauma from the ablation to the implant.

In conclusion, cryoablation is an effective treatment for desmoid tumors. This case report suggests that cryoablation can be performed safely when silicone breast implants are within the expected zone of ablation. Silicone breast implants may not be a deterrent to cryoablation of desmoid tumors.
